# Supplementary material for: Four-party evolutionary game analysis of enterprise environmental behavior
Source: PLoS One. 2024 Oct 9;19(10):e0310280. doi: 10.1371/journal.pone.0310280 (PMC11463782; doi:10.1371/journal.pone.0310280)
Supplement: S1 File — (DOCX) [file pone.0310280.s001.docx]

**Supporting Information**

1. Parameter setting

This paper uses MATLAB software to conduct numerical simulations on the evolutionary trajectory of each game party, which can more intuitively show the key elements that influence the evolution process and results of multi-party games. The parameters are set as follows:

(1) The enterprise enters the countryside, facing environmental pollution in the production process, it will purchase pollution treatment facilities or choose green production approaches when operating in a standardized manner, and the cost is $C_{x1}$=10, and bring positive utility $R_{z}$=10 to farmers with environmental awareness.

(2) When the enterprise chooses nonstandard operation, the operating cost is $C_{x2}=$2.

(3) If the village committee dutifully exhorts the enterprise’s nonstandard business behavior, it will pay $C_{q}$=4, and the enterprise obeys the advice and adds $F1=8$ to the investment in environmental protection facilities.

(4) The regulatory cost of the grass-roots government is $C_{y}$=12. For enterprises that do not operate in a standardized manner, the grass-roots government will impose a fine of $F2=15$ once it is found. A penalty $F4=6$ will be charged to the village committee that irresponsibly handles the environmental damage of enterprises simultaneously.

(5) The continuous nonstandard operations of enterprises will bring losses to farmers and society if $N_{z}=8$ and $N_{y}=15$. The negative effect of farmers with environmental awareness on the village committee who do not do their duty is $N_{q}=5$, and the reputation loss of enterprises is $R_{x}$=6. The probability that farmers choose to protect rights is $\beta=0.1$, the economic compensation obtained is $F3=7$, and the recovered social loss is $T_{y}=10$.

(6) The initial strategies of each player are $x=0.4$, $y=0.3$, $z=0.2$, $q=0.3$.
