# Supplementary material for: Four-party evolutionary game analysis of enterprise environmental behavior
Source: PLoS One. 2024 Oct 9;19(10):e0310280. doi: 10.1371/journal.pone.0310280 (PMC11463782; doi:10.1371/journal.pone.0310280)
Supplement: Attachment — Submitted filename: Response to Reviewers.docx [file pone.0310280.s002.docx]

**Response to Reviewers**

On behalf of all the contributing authors, I would like to express our sincere appreciation for the editor and reviewers’ constructive comments concerning our article. These comments are all valuable and helpful for improving our writing. Point-by-point responses to the excellent editor and two lovely reviewers are listed below this letter.

Reviewer #1

| Dear Professor, thank you for your severe and constructive comments. Those comments are all valuable and very helpful for revising and improving our article, as well as the importance of guiding significance to our research. | | |
| --- | --- | --- |
| **No.** | **Comments** | **Response** |
| 1 | Please explain equations in Table two and could you extract it. | The authors add explanation for Table two. |
| 2 | Please display the meaning of each symbol in the paper. For example, the symbol U in Equation one was not presented. | The authors add explanation for symbol of equations. |
| 3 | It’s not obvious how did you extract equations 1, 2, 3, 4, please  By the same way, you should explain the other equations in the study. | The authors add explanation for equations in the paper. |
| 4 | In all figures, please don’t display the explanation in Chinese language. Explain all what to say in English. | The authors revise all figures in English language. |

Once again, thank you very much for your comments and suggestions. We appreciate the Editors/Reviewers’ warm work earnestly and hope the correction will meet with approval.
